# A reply: Wrong drug problem continues

**DOI:** 10.4103/0019-5049.63644

**Published:** 2010

**Authors:** Vijaya Ramaiah

**Affiliations:** Consultant Anaesthesiologist, East Surrey Hospital, Surrey and Sussex NHS Trust, United Kingdom

Sir,

This is in response to the article "The Wrong Drug Problem Continues" by Singh *et al*.[1] published in the June issue of IJA. Thank you for the pertinent and most important issue which all the anaesthetic fraternity face in our routine practice.

In 1999, the Institute of Medicine called for a halving of error in health care within 5 years.[2] Numerous other authoritative calls for improved safety have been made since, including legislative moves by the Congress and the Food and Drug Administration. Despite this, the vast majority of drugs used in health care continue to be administered by traditional error-prone means, and drug error remains a hazard to patients everywhere. The problem is of particular concern in anaesthesia, where large numbers of potent drugs are given, often in rapid sequence. Perhaps, what is now required to further reduce error in drug administration is a more sophisticated approach, involving a better understanding of the nature of human error itself and better compliance in the adoption of safety procedures and systems.

The important components of safe anaesthetic practice identified include organisational structure with strategic control of healthcare delivery, teamwork and leadership, evidence-based practice, proficiency, continued professional development of all staff, and well-embedded incident reporting and adverse events' disclosure systems. In our quest for the safest possible health care, there is a need for prospective observational multidisciplinary (anaesthetists and human factors specialists) studies as distinct for retrospective reports of adverse events. There is also need for research to establish the ideal system architecture for anonymous reporting of near miss and no harm events in anaesthetic practice.

There is an increasing recognition that medication errors are causing a substantial global public health problem, as many result in harm to patients and increased costs to health providers.[3] However, study of medication error is hampered by difficulty with definitions, research methods and study populations. Few doctors are as involved in the process of prescribing, selecting, preparing and giving drugs as anaesthetists, regardless of whether their practice is based in the operating theatre, critical care or pain management. Anaesthesia is now safe and routine, yet anaesthetists are not immune from making medication errors and the consequences of their mistakes may be more serious than those of doctors in other specialties. Steps are being taken to determine the extent of the problem of medication error in anaesthesia. New technology, theories of human error and lessons learnt from the nuclear, petrochemical and aviation industries are being used to tackle the problem.

Safe drug administration is the responsibility of health care providers and drug regulatory authorities. Medication errors are an inevitable consequence of the human condition; they occur even among the most conscientious medical professionals. If health care professionals do not demand, on behalf of their patients, reasonable safeguards to reduce the likelihood of medication errors, no one else will. Health care professionals should report their concerns regarding the quality, safety, performance or design of products used in their practice to the authorities. These authorities need to identify the underlying causal mechanisms and develop strategies to prevent their recurrence. This information is then shared with industry and appropriate government agencies.

Proper IV administration should follow the five "rights" of medication administration to avoid medication errors: be sure it is the right patient, the right drug, the right dose, the right time, and the right route before giving any medication.
